# Screening abstinent bariatric surgery patients for Behavioural Addictions using MMPI-2 data

**DOI:** 10.1192/j.eurpsy.2025.430

**Published:** 2025-08-26

**Authors:** F. Császár, R. Jávor, I. B. Bálint

**Affiliations:** 1 Psychiatry, Vas County Markusovszky University Teaching Hospital, Szombathely; 2 Faculty of Humanities and Social Sciences, University of Pécs, Pécs; 3 Department of Urology, Kanizsai Dorottya Hospital, Nagykanizsa, Hungary

## Abstract

**Introduction:**

Eating disorders comprise various conditions yet do not cover chronic overeating that may result in extreme obesity. Binge eating disorder with chronic somatic effects is not included in DSM-V; behavioural addictions do not comprise chronic overeating either. Neither do impulse control disorders. There are no actual screening tools for chronic overeating, and research is scarce on its chronic psychological effects

**Objectives:**

This research aims to find the distinctive psychometric characteristics of addiction using MMPI-2 data taken from patients who underwent gastric surgery due to high-risk obesity or moderate-risk obesity with alarming comorbidities.

**Methods:**

This study employed a consecutive patient cohort to evaluate complication rates and the efficacy of Single-Anastomosis duodeno-ileal bypass with Gastric plication (SADI-GP). Patient recruitment commenced in October 2018 and ceased in June 2019. The process involved preoperative assessment, surgery, and several postoperative follow-up appointments at 1, 3, 6, and 12 months. The Minnesota Multiphasic Personality Inventory (MMPI-2) was administered during the 12-month follow-up. Participants aged between 18 and 65 years were included in the study, with body mass indexes (BMIs) exceeding 40 for individuals without comorbidities related to morbid obesity, and exceeding 35 for those with comorbidities related to morbid obesity, particularly related to glucose metabolism.

MMPI-2 scales previously confirmed to be related to SUD were analyzed, and common psychological comorbidities of SUD were searched for using these scales

**Results:**

High scores on MAC-R, AAS, and APS scales are well-represented in the sample (Table 1).

The sample includes a high number of high scorers on Rc4 and a moderately high number of high scorers on Rc9 (Table 2).

Elevated individual scale scores form dual or triplet peak settings in the MMPI-2 results and may describe certain conditions, like SUD. The majority of the subjects showed SUD-like personality settings (Figure 1). This study is constrained by limitations about sample size, a dropout rate exceeding expectations, stringent exclusion criteria, male-to-female ratio, short-term results, and the absence of longitudinal data on psychological characteristics.

**Image 1:**

**Figure:**
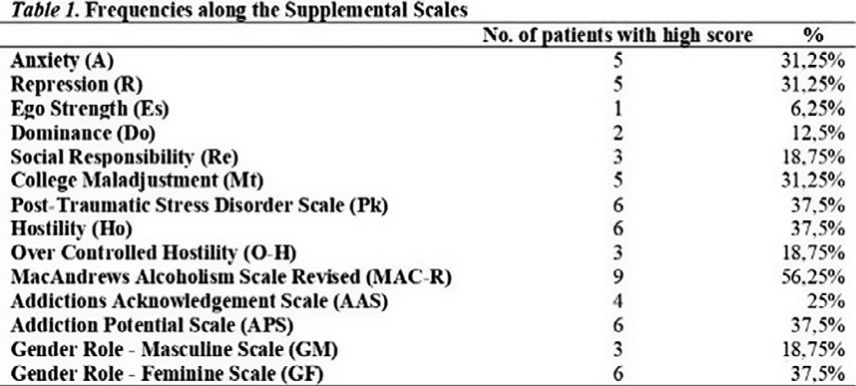

**Image 2:**

**Figure:**
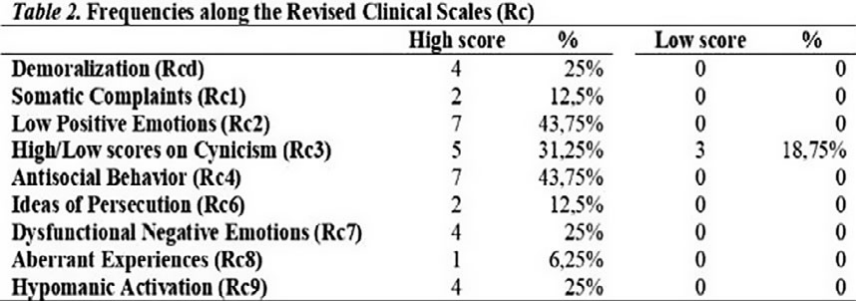

**Image 3:**

**Figure:**
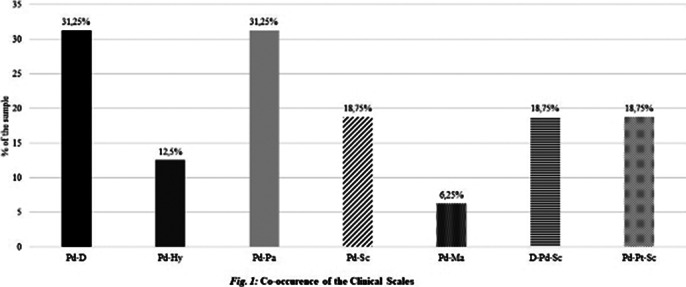

**Conclusions:**

We found the MacAndrews Revised (MAC-R) scale strong, with AAS and APS as intermediate indicators for non-substance-based behavioural addiction in our sample (Table 1). RC4 also seems to be a strong indicator (Table 2), along with Pd-D and Pd-Pa peaks (Figure 1).

**Disclosure of Interest:**

None Declared

